# High-Throughput SuperSAGE for Digital Gene Expression Analysis of Multiple Samples Using Next Generation Sequencing

**DOI:** 10.1371/journal.pone.0012010

**Published:** 2010-08-06

**Authors:** Hideo Matsumura, Kentaro Yoshida, Shujun Luo, Eiji Kimura, Takahiro Fujibe, Zayed Albertyn, Roberto A. Barrero, Detlev H. Krüger, Günter Kahl, Gary P. Schroth, Ryohei Terauchi

**Affiliations:** 1 Iwate Biotechnology Research Center, Kitakami, Japan; 2 Gene Research Center, Shinshu University, Ueda, Japan; 3 Illumina, Inc., Hayward, California, United States of America; 4 Department of Anatomy, Iwate Medical University, Morioka, Japan; 5 Centre for Comparative Genomics, Murdoch University, Perth, Australia; 6 Institute of Medical Virology, University Hospital Charite, Berlin, Germany; 7 Molecular BioSciences, Biocentre, University of Frankfurt am Main, Frankfurt, Germany; University College London, United Kingdom

## Abstract

We established a protocol of the SuperSAGE technology combined with next-generation sequencing, coined “High-Throughput (HT-) SuperSAGE”. SuperSAGE is a method of digital gene expression profiling that allows isolation of 26-bp tag fragments from expressed transcripts. In the present protocol, index (barcode) sequences are employed to discriminate tags from different samples. Such barcodes allow researchers to analyze digital tags from transcriptomes of many samples in a single sequencing run by simply pooling the libraries. Here, we demonstrated that HT-SuperSAGE provided highly sensitive, reproducible and accurate digital gene expression data. By increasing throughput for analysis in HT-SuperSAGE, various applications are foreseen and several examples are provided in the present study, including analyses of laser-microdissected cells, biological replicates and tag extraction using different anchoring enzymes.

## Introduction

Next Generation Sequencing (NGS) technology is revolutionizing the way we study biological problems [1], [2]. The four main NGS platforms already allowed the de novo sequencing of a multitude of bacterial, archaeal, fungal, plant and animal genomes, and this development is spurred on by the rapid development of efficient sequence assembly software tools like “Velvet” [3]. NGS also enables rapid whole genome re-sequencing without the cloning and costs associated with conventional Sanger sequencing, so that SNP identification can be enormously facilitated and catalyzes genetic studies in a wide array of organisms [4].

Another important application of NGS is gene expression analysis. Traditionally, sequencing-based gene expression was approached by Expressed Sequence Tag (EST) analysis [5], Serial Analysis Gene Expression (SAGE) [6], LongSAGE [7], SuperSAGE [8] and Massively Parallel Sequencing Signatures (MPSS) [9]. Until quite recently, microarray technology has dominated gene expression profiling. However, the development of NGS technologies totally changed the way we study gene expression, the structure of the transcriptome, and RNA processing. It is clear that sequencing-based transcriptome analysis in many ways is superior to microarrays, since sequencing-based method is digital, highly accurate, and easy-to-perform, whereas the microarray_-_generated data are analog and less accurate, and their acquisition requires specific probe and array designs. Therefore, some have predicted that microarrays will soon be replaced by sequencing-based digital gene expression analysis [10]. Application of NGS to gene expression analysis has catalyzed the development of techniques like Digital Gene Expression TAG (DGE-TAG), DeepSAGE [11], [12] and RNA-Seq [13], [14]. However, the standard DGE-TAG assay provides relatively short tag reads (21 bp) which sometimes leave tag-to-gene annotation more difficult, and RNA-Seq requires a large amount of sequence reads to fully cover the dynamic range and to provide a truly quantitative gene expression profiling. Therefore, a reliable protocol of tag-based gene expression profiling based on sequencing of longer tag fragments is highly desirable. Since the cost of sequencing continues to decay, it is additionally important to develop an indexing protocol that permits to analyze multiple samples in a single sequencing run, thereby increasing sample throughput per run, and reducing the costs per sample.

In this report, we introduce a protocol for NGS-based SuperSAGE profiling that is adapted to the simultaneous analysis of multiple samples and coined High-Throughput (HT-) SuperSAGE. For multiplexing different samples in a single sequence run and a single lane on the Illumina Genome Analyzer, we use index sequences (bar-coding). Here, we illustrate this method to demonstrate its sensitivity, reproducibility and accuracy. Finally, we portray some of the possible applications of this advanced technology, with examples from several different species.

## Results

### HT-SuperSAGE protocol for sample multiplexing

**Figure 1:**
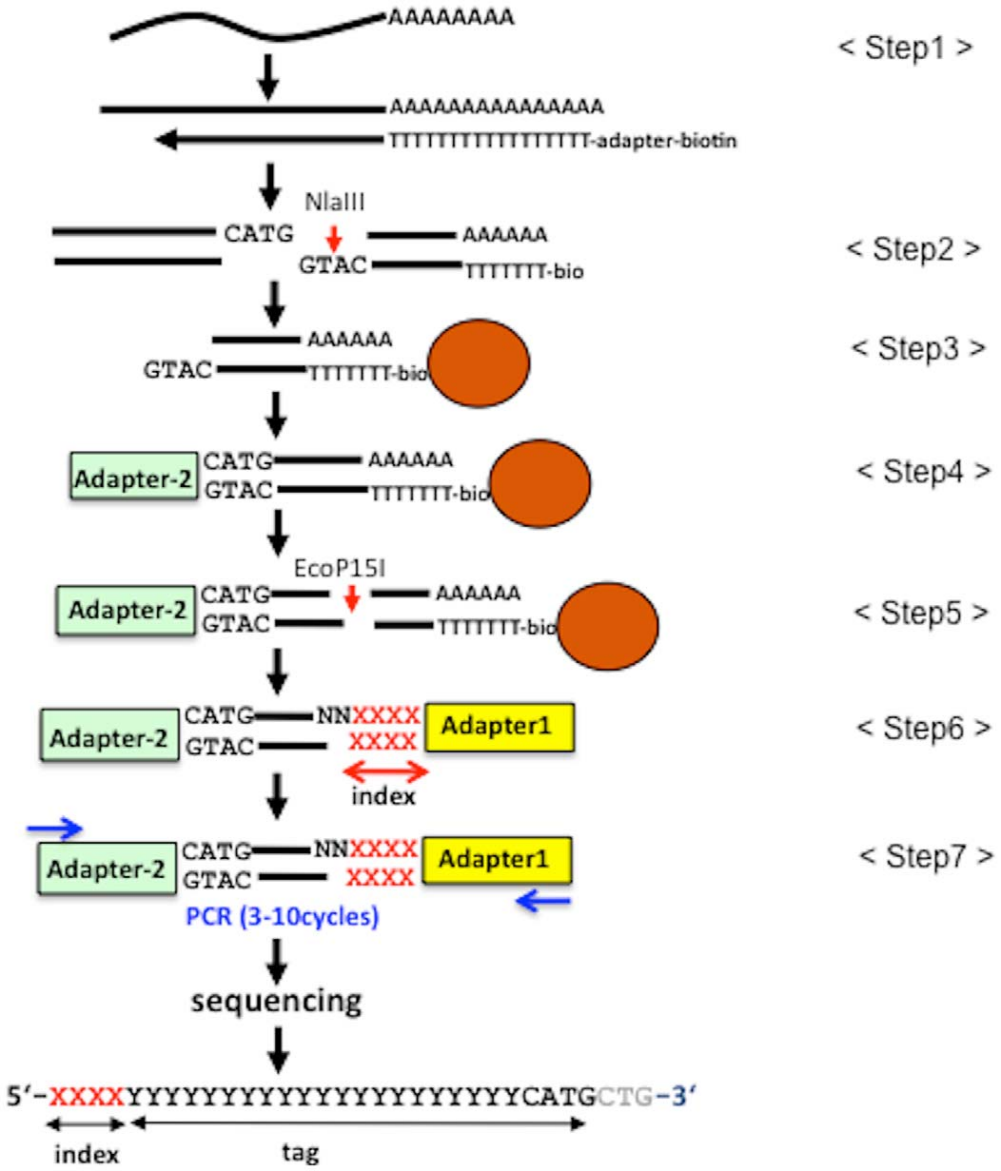
Scheme of high-throughput SuperSAGE. Details of the experimental procedure are described in Results and Material and Methods.

The workflow of our experimental procedure from RNA to the sequencing of high-throughput SuperSAGE (HT)-SuperSAGE tags is depicted in Figure 1. This method mimics the original SuperSAGE protocol [8] up to the step where the 26-bp tag fragments are cut from double-stranded cDNAs (Figure 1, step 5). However, after this step we do not form “ditags” comprising two tags in inverted orientation as described in the original SuperSAGE protocol [7]. Instead, two adapters are ligated to each end of a single tag (Figure 1, step 6), and the “adapter-tag” fragments are amplified by PCR for a limited number of cycles (Figure 1, step 7).

Increasing sequencing reads, rare transcripts could be obviously identified in Ht-SuperSAGE. Also, according to Asmann *et al.* [15], increasing sequencing reads (0.5 to 96 million) in DGE analysis, dynamic range of its profiling data is proportionally expanded. However, more than millions of tags are not always essential for every study, and simultaneous analysis of multiple different samples might be required as an application of gene expression analysis. Therefore, it is imperative to multiplex samples (libraries) to increase sample throughput per run and reduce the cost of analysis per sample. For analyzing multiple samples in a single sequencing run, we employ an indexing (bar-coding) system. Adapter fragments harboring different index sequences are ligated separately to 26-bp tag fragments derived from different biological samples. Adapter-tag fragments from different libraries are pooled and sequenced together. Later, the sequence reads are separated *in silico* according to their index sequences. We have designed 4-base oligonucleotides located at the end of adapter-1 (just downstream of the sequencing primer site) as the index. Therefore, the first four bases in a sequence read encode the index, and the subsequent 26–27 nucleotides are tag sequences derived from mRNA, including the recognition site of the anchoring enzyme (Figure 1, bottom).

### Preparation of HT-SuperSAGE libraries

We prepared total RNAs from 24 different tissue samples derived from three different organisms (rice, zebra fish, *Arabidopsis*; Table 1). In 20 of the samples, 5µg total RNA was used for cDNA synthesis. Four of the samples (sample g, h, i and j; Table 1) represented RNAs purified from two fungal pathogen-infected rice cells, rice pollen and rice anther wall cells, which were isolated by laser microdissection, LMD [16]. For the rice mature leaf sample, cDNA was divided into three tubes, and three different indexed adapters were ligated to each (samples a, b, and c; Table 1) aiming to evaluate the influence of PCR-amplification cycle number on expression profiles as described below.

**Table 1:** Summary of all the analyzed samples.

Sample code	Sample name	Index seq	Number of total tags	Number of unique tags	Number of non-singleton tags
a	rice leaf(3-cyclePCR)	GCCC	353,524	51,314	18,956
b	rice leaf (5-cycle PCR)	GCCA	517,891	69,055	24,706
c	rice leaf (10-cycle PCR)	GCCT	295,439	45,846	16,790
d	5 PCR rice seedling-1	GCCG	367,798	74,506	22,902
e	5 PCR rice seedling-2	GCAC	483,836	78,205	26,379
f	5 PCR rice seedling-3	GCAA	388,658	71,455	23,549
g	*M.grisea*-infected rice cells (30h after inoculation)	GCAT	729,542	91,311	31,954
h	*M.grisea*-infected rice cells (48h after inoculation)	GCAG	383,022	69,448	21,736
i	rice pollen cells	GCTC	348,370	67,317	24,002
j	rice anther wall tissue	GCTA	301,118	69,498	22,162
k	rice mutant seedling (l*m1*)	GCTT	537,192	92,133	29,377
l	*M.grisea*-infected rice leaf	GCTG	420,326	84,014	27,586
m	CM552 seedling (allele of *lm1*)	GCGC	311,581	63,781	21,120
n	SG0807 seedling (allele of *lm1*)	GCGA	321,433	67,868	21,817
o	rice germinating seed (c.v.Dunghan shali at low temp)	GCGT	489,818	91,408	28,873
p	rice germinating seed (c.v. Kakehashi at low temp)	GCGG	92,410	29,779	7,214
q	rice germinating seed (c.v.Dunghan shali ,submerged)	GACC	394,443	65,955	22,510
r	rice germinating seed (c.v. Kakehashi ,submerged)	GACA	784,859	114,234	36,423
u	zebrafish embryo 10.5h after fertilization	GAAC	484,471	85,922	26,788
v	zebrafish embryo 12h after fertilization	GAAA	665,730	106,953	30,791
w	zebrafish embryo 13.5h after fertilization	GAAT	582,844	113,931	29,656
x	zebrafish embryo 15h after fertilization	GAAG	620,402	105,314	30,772
y	zebrafish embryo 16.5h after fertilization	GATC	466,332	82,625	24,741
z	wild type rice (cv. Sasanishiki) leaf	ACCC	530,176	71,640	23,806
ex1	*Pex33*-overxpressing rice leaf	TCCA	456,718	71,591	25,389
s1	Arabidopsis leaves (NlaIII; CATG)	GACT	399,106	67,949	23,903
s2	Arabidopsis leaves (DpnII; GATC)	GACT	260,326	51,671	18,669
s3	Arabidopsis leaves (BfaI;CTAG)	GACT	233,475	43,168	16,069
t1	Arabidopsis stems (NlaIII; CATG)	GACG	303,549	63,738	23,318
t2	Arabidopsis stems (DpnII; GATC)	GACG	351,677	66,435	25,207
t3	Arabidopsis stems (BfaI;CTAG)	GACG	239,537	45,630	18,064
	Total		13,115,603		

Double-stranded cDNA was digested with the anchoring enzyme NlaIII (recognition site: 5′-CATG-3′), and tag fragments were isolated from the NlaIII site closest to the poly-A tail of cDNA. For the two *Arabidopsis* tissue samples (samples s and t; Table 1) we used DpnII and BfaI in addition to NlaIII to test how different anchoring enzymes affect the final transcription profiles. Equal amounts of each cDNA were separated into three tubes and digested with NlaIII, DpnII and BfaI, so that tags could be extracted from three different positions (5′-CATG-3′, 5′-GATC-3′ and 5′-CTAG-3′,respectively) that are closest to the poly-A tail in the cDNA sequences.

Adapter-1 fragments, ligated to EcoP15I-digested fragments, contain a 4-bp index (bar-code) sequence for library identification. In the present study, a total of 27 adapters with different index sequences were prepared and allocated to individual libraries (Table 1). Equal amounts of PCR products (123–125bp) from all the libraries were mixed in one tube, and the resulting DNA pool was sequenced using three lanes in a flow-cell of the Illumina Genome Analyzer GA_II_.

### Tag sequence retrieval

In total, 16,057,777 sequence reads (35-bases) were obtained by sequencing. As described, the first four bases are index sequences for library discrimination, and therefore the actual tag sequence starts at the fifth base in the read and terminates at the anchoring restriction enzyme sites (NlaIII, DpnII or BfaI; Figure 1, bottom). After removing incomplete sequences (without index sequences and/or anchoring enzyme sites), 13,142,905 reads were selected. Using a script written in Perl, these sequence reads were separated into 27 groups based on index sequences, and then tag sequences were extracted and their frequencies counted in each group. Sequence reads from *Arabidopsis* samples (samples s and t; Table 1) were further classified into three subgroups each on the basis of the anchoring enzymes (NlaIII, DpnII and BfaI).

Since the distance between recognition and cleavage sites is not uniform for EcoP15I, tags with various sizes are frequently produced. The distribution of tag lengths in a selected library (Figure S1; rice leaf sample; sample c) shows that the most abundant tag length in these libraries is 27-bases (66%), followed by tags of 26-bases (25%). In the following analysis, we decided to extract 26-bp sequences from all the reads to represent SuperSAGE tag.

### Identification of libraries by index sequences

After classification of sequences by index and anchoring enzyme sites, the 26-bp tag profiling data were obtained from all the 31 samples (Table 1). The number of tags varied from 92,410 (sample q) to 729,542 (sample g) with an average of 423,964 tags per sample. The top ten most abundantly expressed tag sequences in each group were BLASTed against non-redundant (nr) nucleotide sequences of Genbank. Most of them completely matched genome or cDNA sequences from the species of origin (Table S1). As expected, tag sequence data from different biological samples were properly discriminated by the 4-bp index sequences.

However, it is still possible that single-base errors in the 4-bp index might cause contamination of tags from different samples. To evaluate the frequency of contamination we focused on an index sequence GACT (sample s1; *Arabidopsis thaliana* tissue), which can change to GCCT, GCTT, GACC or GACA (sample c, k, q or r; *Oryza sativa* tissues), respectively, by a single-base error. Errors in these index sequences could be a potential cause, when the species identified by the tag sequences does not correspond to the species represented by the index sequence. In each sample, the most abundant 5,000 tags were applied to a BLAST search against UniGene data of *Oryza* sativa and *Arabidopsis thaliana*. In sample s1 (*Arabidopsis*), 8 tags matched *Oryza sativa* genes, while no *Arabidopsis* tags were found in data of samples c, k, q or e, suggesting that contamination due to errors in the index sequences indeed occurs, albeit at a low level (data not shown). In fact, their frequency was less than 0.2% of total analyzed tags, indicating that they do not cause distortion in the gene expression profiles.

### Influence of PCR cycle numbers on expression profiling

**Figure 2:**
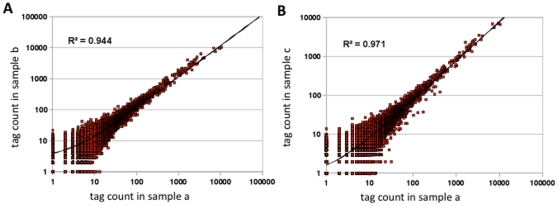
Influence of PCR cycle numbers on tag abundance. Tags from mature rice leaf samples were ligated to adapter-2 sequences, separated into three tubes, and then three differently indexed adapters-1 (a, b and c, respectively) were ligated. The adapter-1 ligated fragments were PCR amplified for three (sample a), five (sample b) and ten cycles (sample c), and subsequently directly sequenced. After sequencing, tag abundance data was obtained for each sample. Individual tag counts are plotted for sample a versus b (A), and sample a versus c (B). Correlation coefficient in each plot is shown as inset (R^2^), and regression line is indicated as curved line due to the plot on logarithmic scale.

Since PCR amplification of adapter-tag fragments may cause a distortion in transcript profiles due to preferential amplification of a subset of tags, we evaluated how the number of PCR cycles affects tag profiles. As described above, adapter-2 ligated tag fragments from rice mature leaf were separated into three tubes and adapter-1 fragments with different indexes (a, b and c) were ligated. These ligation products, with the a, b and c index sequences were amplified for three, five or ten cycles of PCR, respectively. Sequence reads from these PCR products were separated by index sequences and tag abundance data obtained for each sample. Counts of individual tags are plotted for samples a (3-cycles; x-axis) versus b (5-cycles; y-axis) (in Figure 2A, or for samples a (3-cycles; x-axis) versus c (10-cycles; y-axis) in Figure 2B. In both cases, their tag counts showed highly significant correlations (R^2^>0.9), which readily demonstrates that an increase in PCR cycle numbers up to 10 does not cause any significant distortion in the expression profile.

### HT-SuperSAGE versus original SuperSAGE

**Figure 3:**
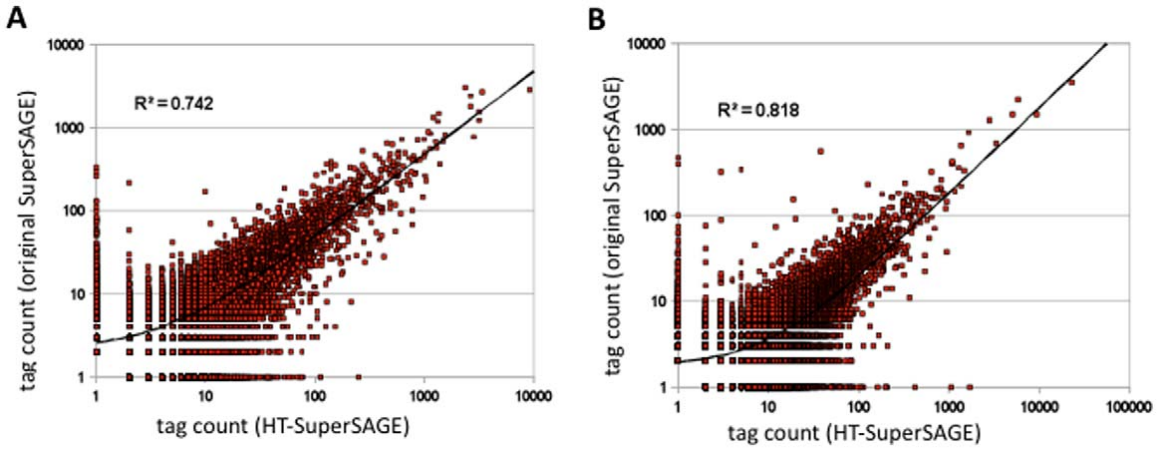
Comparison of tag abundance between HT-SuperSAGE and original SuperSAGE. Synthesized cDNAs from rice seedling RNA (sample f) and *M. grisea*-infected rice leaf sheath RNA (sample l) were divided into half and both applied to HT-SuperSAGE and original SuperSAGE, respectively, using 454-pyrosequencing. Obtained counts of individual tags from the two methods are plotted (panel A for sample f, and panel B for sample l). Correlation coefficient in each plot is shown as inset (R^2^), and regression line is indicated as curved line due to the plot on logarithmic scale.

Compared to the original SuperSAGE technique, several modifications were introduced into HT-SuperSAGE. In particular, the steps of ditag formation, the PCR conditions and the sequencing method were altered. To compare the tag frequencies obtained by the two protocols, cDNAs from two different samples (rice seedlings; sample f, and *M. grisea*-infected rice leaf sheath; sample l) were divided into half and either applied to Illumina sequencing (HT-SuperSAGE), or 454 pyrosequencing (original SuperSAGE) [17]. In HT-SuperSAGE, adapter-tag fragments were amplified for five PCR cycles. In the original SuperSAGE analysis, ditags were formed by ligation of two adapter-tag fragments, and PCR-amplified for 23 cycles. After sequencing of the ditag PCR products, duplicated ditag sequences were excluded before tag extraction and counting, and thus followed the original SAGE protocol [7]. The total number of tags obtained by these two methods was shown in Table S2. For a comparison of the two protocols, individual tag counts obtained in both are plotted (Figure 3). The tag profiles between HT-SuperSAGE and conventional SuperSAGE were basically similar, although the correlation coefficients were not high (R^2^ = 0.742 for sample f, and 0.818 for sample l).

We recognized that tags containing homopolymer sequences are underrepresented in the original SuperSAGE library sequenced using 454 pyrosequencing (Table S3). Such tags harboring homopolymer stretches frequently carry similar sequences with one or two base changes in the original SuperSAGE data, while such variant sequences were not observed in HT-SuperSAGE (Table S4). BLAST searches suggest that only one tag sequence has a perfect match to the database, and the variants do not. This fact strongly suggests that the variants originated from PCR or sequencing errors. To evaluate the influence of homopolymer or erroneous sequences in the tags, we excluded the tags harboring homopolymer (>5 nucleotides) sequences and the tags, which did not completely match rice or *Magnaporthe* genome sequences. Their correlation coefficients between HT-SuperSAGE and conventional SuperSAGE were improved to 0.799 in sample f and 0.836 in sample l (Figure S2). We conclude from these results that HT-SuperSAGE provides more accurate expression data from tags containing homopolymer sequences than the original SuperSAGE method. In addition, the HT-SuperSAGE protocol does not involve ditag ligation, which obviously bias SAGE results, and, moreover, uses much less PCR cycles (10 or less, compared to 23 cycles for SuperSAGE). We speculate that these differences between HT-SuperSAGE and original SuperSAGE are responsible for the discrepancy in tag counts, shown even after removal of homopolymer-containing and erroneous tags.

### Application of HT-SuperSAGE to biological studies

HT-SuperSAGE handles a large number of samples at low cost, hence recommends itself for an application to various biological studies. We demonstrated that this technique is applicable to expression analyses of tissues placed under different environmental conditions and collected at different time points of organ development, as well as genetic mutants and transgenic plants as exemplified in Table 1. Also, a combination of HT-SuperSAGE and LMD allows to characterize cell-specific gene expression. Apart from these well-accepted applications, we suggest two new applications below, which were impractical in the previous Sanger sequencing-based gene expression analysis.

### Gene expression analysis of biologically replicated samples

Analyses of biologically replicated samples are prerequisite for evaluating whether an identified differential gene expression pattern is in fact a response to a particular treatment, or not. In the current study, shoot tissues were collected from three separate rice plants grown under identical conditions, and served as the biological replicates (sample d, e and f in Table 1). The comparison of tag counts between the replicates e and f showed a highly significant correlation coefficient (R^2^ = 0.9816), while a comparison between samples involving sample d did not (Figure S3). Among these tag profiling data one tag with the sequence CATGACAAGTTTTTGTTAATAATAAT, corresponding to the ribulose-bisphosphate carboxylase activase gene (Os11g0707000 was represented by less counts in sample d (3,291 tags) as compared to the two other replicates (11,544 and 9,234 tags). As shown in the present result, owing to the low cost and high-throughput, HT-SuperSAGE allows taking sufficient number of biological replicates to verify the gene expression profiles.

### Tag extraction using different anchoring enzymes

To test the effect of different anchoring enzymes on the final expression profiles, we employed two restriction enzymes DpnII and BfaI in addition to NlaIII for the isolation of tags from the same cDNA samples. For this experiments we used *Arabidopsis* leaf and stem samples. Obtained tags were mapped to *Arabidopsis* genes for comparing count of each tag for the individual gene (Table S5). If all cDNAs harbored recognition sites of the three anchoring enzymes equally, then tag counts for each gene should be similar among the tags obtained by the three anchoring enzymes. Actually, it was frequently observed that tag counts for particular genes were undetectable or significantly less for one or two anchoring enzymes. In order to estimate the frequency of these “missing transcripts”, we focused on the 1,000 most abundantly expressed genes in *Arabidopsis* leaf and stem based on the present HT-SuperSAGE data (Table S5). The abundance of transcripts from each gene was represented by the average of normalized tag counts (tag counts per one million tags) among all NlaIII, DpnII, and BfaI tags, respectively. A “missing transcript” was defined as a tag, whose count was less than 10% of transcript abundance (average from the sum of tags obtained from the three enzymes). It was observed that missing transcripts in NlaIII or DpnII tags comprised approximately 7–8% of expressed genes (Table 2). BfaI tags were more frequently missing than others (around 19% of expressed genes). There was no significant difference in the frequency of missing transcripts between *Arabidopsis* leaf and stem samples. Also, individual missing transcripts from each gene were equally distributed between two tissue samples, indicating the good reproducibility of these result. This “missing” transcript phenomenon is undoubtedly a consequence of differences in the location and presence/absence of the restriction enzyme recognition site in the transcript.

**Table 2:** Missing transcripts from abundantly expressed *Arabidopsis* genes studied by three anchoring enzymes (NlaIII, DpnII, BfaI) in leaf and stem tissues.

	Number of missing tags*		
	NlaIII	DpnII	BfaI
Leaf (sample s)	64	81	188
Stem (sample t)	76	59	205

*Missing transcripts in 1000 of the most abundantly expressed genes in each *Arabidopsis* leaf or stem tissue samples (see text).

## Discussion

Here we present an easy-to-perform protocol for a new technique called HT-SuperSAGE, which consists of a modified SuperSAGE protocol with the Illumina Genome Analyzer next generation sequencing platform. The major advantage of HT-SuperSAGE over other similar techniques is that 26-bp tag sequences can be isolated from each transcript, which is so far the longest tag sequence obtained from a defined position of transcripts. These 26-bp tags allow a much better and unambiguous tag-to-gene identification, which is just not possible with shorter tags [8]. Importantly, in HT-SuperSAGE we incorporated an indexing scheme to multiplex the libraries and increase the throughput, which actually allowed an analysis of 31 libraries in three eighth of the capacity of a single Illumina GAII sequencing run (Table 1). It should be easy to analyze >100 libraries in a single sequencing run by simply employing a corresponding number of different index sequences. Considering to the power of sequencing technology, huge number of tags (more than ten million) could be easily analyzed. However, we suggested analysis of 0.5–1 million tags per a sample as routine studies, since 20–30 thousands of unique non-singleton tags could be identified in this scale of analysis, which were expected to cover most of expressed genes in eukaryotes. The high throughput and low costs of HT-SuperSAGE now allows the analysis of biological replicate (i.e. multiple) samples (Figure S3), which was not easy in the previous sequence-tag-based transcriptome analyses. Technically, there is no problem in increasing the number of replicates, although their data analysis procedure should be considered in further studies. Analytical scale was flexible in HT-SuperSAGE, since number of samples for multiplexing and sequencing reads for each sample could be changed as we like. As described in original SuperSAGE method, it was applicable to any eukaryotic life organisms [8]. In view of these advantages we propose that the performance and potential of HT-SuperSAGE is comparable, if not superior to microarray techniques.

In the original SuperSAGE procedure, any duplicated ditag sequence was excluded as a PCR amplification bias, which permitted to maintain an accuracy of transcript profiles [8]. In the present advanced method, ditag fragments are no longer produced so that this strategy did not have to be involved. In order to minimize distortion in the expression profiles due to the amplification bias, we reduced the number of amplification cycles as much as possible, although in the end the effect of PCR on tag abundance was minimal. We proved that 10 PCR cycles yielded enough adapter-tag DNA (at least several hundred pico gram per a PCR reaction, starting from 5µg total RNA), and that the tag profiles were not different for 3, 5 and 10 PCR cycles, respectively, if high-fidelity DNA polymerase was used (Fig 2). In summary, we propose that PCR amplifications of up to 10 cycles will not cause any detectable errors in the final HT-SuperSAGE profile data.

To evaluate the impact of different anchoring enzymes on the final expression profiles, 26-bp tags were extracted from different positions within the cDNAs using the three different anchoring enzymes NlaIII, DpnII and BfaI. Most of the tags in previous SAGE [6], LongSAGE [7] or SuperSAGE [8] experiments were derived from NlaIII sites in cDNA. To the best of our knowledge, the present report introduces the first comprehensive experimental comparison of large-scale tag data from the same cDNA pool using three different anchoring enzymes. Our results show that neither of the three enzymes can cover all the transcripts. Even NlaIII, the most commonly used enzyme, missed 7–8% of the transcripts. BfaI failed to recover ∼20% of transcripts, and is therefore not suitable for HT-SuperSAGE. It is probable that a low frequency of BfaI recognition sites in genes may cause this failure. According to *in silico* sequence data analysis of Arabidopsis RefSeq database, within 35,286 genes, 2,000 genes (5.7%) and 1,601 genes (4.5%) did not have NlaIII and DpnII sites, respectively. Number of genes without BfaI site was 4,733 (13.4%). These frequencies were similar to the present experimental results. Pleasance *et al.* (2003) already reported that genes without BfaI sites apparently outnumbered those without NlaIII or Sau3AI (DpnII) restriction motifs both in *D. melanogaster* and *C. elegans* [18]. This phenomenon is consistent with our experimental results in *Arabidopsis* expressed genes. Since the percentages of missing transcripts were similar in both NlaIII- and DpnII -derived tag populations (7–8% of expressed genes), >99% ( = 1–0.08^2^) of expressed genes could theoretically be monitored by employing these two anchoring enzymes.

As described above, the HT-SuperSAGE protocol was developed for simultaneously analyzing digital gene expression of many different samples using the Illumina Genome Analyzer platform. Obviously, this advanced protocol is also compatible with other next generation sequencers with minor modifications in adapter design. We anticipate that HT-SuperSAGE-based transcriptome analysis will become one of the most powerful applications of the next-generation sequencing (NGS) technology.

## Materials and Methods

### RNA preparation

Total RNA was extracted from the tissues listed in Table 1 (rice, *Arabidopsis* and zebrafish). Rice seedlings (cv. Kakehashi in sample a to f, *lm1* in sample k, CM552 in sample m, SG0807 in sample n, cv. Sasanishiki in sample z and *Pex33*-overexpressing rice [19] in sample ex1) were grown at 28°C for 30 days after seed germination. For preparing infected fungal infected tissues, *M.grisea* conidia were inoculated on the leaves of rice (cv. Sasanishiki) seedlings grown for 30days. Pollen and anther wall tissues were collected from panicles before heading of rice plants (cv. Sasanishiki). Rice germinating seeds were prepared by sowing rice seeds at 15°C or under submerged condition at 28°C. Zebrafish (*Tg(fli1:EGFP)y1*; *albino b4/b4*) embryos were collected at 10.5–16.5 h after fertilization. *Arabidopsis* leaf and stem tissues were collected from mature plants grown at 22°C for 2 months. For cDNA synthesis, 5–10µg total RNA per tissue sample was used, except for laser microdissected tissues.

### RNA amplification

For RNA extraction of minute tissue samples, tissues were once fixed with ethanol-acetate (3∶1) solution, and the solution subsequently substituted for PBS (Phosphate Buffered Saline) with 10% sucrose [16]. Fixed tissues were embedded in OCT (optimal cutting temperature) comp0und (Sakura Seiki) for cryosectioning. After freezing of the OCT compound with tissues, 10–14µm cross-sections were prepared using a cryostat (Zeiss), and mounted on glass slides coated with a membrane film for dissection. The glass slides were then fixed by rinsing with 70% ethanol, subsequently dried, and laser microdissection (LMD, PALM-MB; Zeiss) applied. From tissues or cells collected by LMD, RNA was extracted using the PicoPure RNA isolation kit (Arcturus).

The first round of RNA amplification followed the “Eberwine” procedure [20], which amplifies antisense RNA from cDNA templates using T7 RNA polymerase. In the second round, we employed an improved version of sense-strand RNA amplification method for SAGE [21]. Extracted RNA was once amplified with the TargetAmp 1-Round aRNA Amplification Kit (EPICENTRE). From the amplified antisense RNA, double-stranded cDNA was synthesized using random hexamers and biotinylated oligo-dT. The cDNA, after NlaIII digestion, was captured on streptavidin-coated magnetic beads. Then a T7 linker was ligated to the digested cDNA on the beads, and RNA amplified from cDNA by *in vitro* transcription with T7 RNA polymerase [21]. The resulting RNA (around 10 µg) was the template for cDNA synthesis and tag extraction. Before the bulk tag extraction process was started, rice actin cDNA was first PCR amplified for a test of the procedure.

### Adapter preparation

For adapter-2, the two oligonucleotides (5′-CAAGCAGAAGACGGCATACGATCTAACGATGTACGCAGCAGCATG-3′ and 5′-CTGCTGCGTACATCGTTAGATCGTATGCCGTCTTCTGCTTG- amino-3′), and for adapter-1, the two oligonucleotides (5′-ACAGGTTCAGAGTTCTACAGTCCGACGATCXXXX-3′ and 5′-NNXXXXGATCGTCGGACTGTAGAACTCTGAACCTGT-amino-3′; XXXX encodes variable index sequences.) were synthesized and annealed.

Adapter-2Dpn was prepared by annealing the two synthetic oligonucleotides (5′-CAAGCAGAAGACGGCATACGATCTAACGATGTACGCAGCAG-3′ and 5′-GATCCTGCTGCGTACATCGTTAGATCGTATGCCGTCTTCTGCTTG- amino-3′),and adapter-2Bfa by the annealing oligonucleotides (5′-CAAGCAGAAGACGGCATACGATCTAACGATGTACGCAGCAGC-3′ and 5′-CTAGCTGCTGCGTACATCGTTAGATCGTATGCCGTCTTCTGCTTG- amino-3′).

### Tag extraction and preparation of sequencing templates

Double-stranded cDNA was synthesized using the biotinylated adapter-oligo dT primer (5′-bio-CTGATCTAGAGGTACCGGATCCCAGCAGTTTTTTTTTTTTTTTTT-3′). Purified cDNA was digested with anchoring enzymes (NlaIII, DpnII or BfaI), resulting fragments were bound to streptavidin-coated beads (Dynabeads streptavidin M-270), and non-biotinylated cDNA fragments were removed by washing. Adapter-2 (or adapter-2Dpn, or adapter-2Bfa) was ligated to cDNA fragments on the beads and after washing digested with EcoP15I. EcoP15I-digested and released fragments (adapter-2- tags) were ligated to adapters-1 with defined index sequences for sample identification.

Tags sandwiched between two adapters were amplified by PCR using PhusionHigh polymerase and GEX primers (5′-AATGATACGGCGACCACCGACAGGTTCAGAGTTCTACAGTCCGA-3′ and 5′-CAAGCAGAAGACGGCATACGA-3′). The PCR regime consisted of 98°C for 1min, 3–10 cycles at 98°C for 30sec, and 60°C for 30sec. Eight tubes from this PCR amplification (each 15µl) were pooled and concentrated PCR products using MinElute reaction purification kit (Qiagen) were run on an 8% non-denaturing polyacrylamide gel. After staining with SYBR green (Takara Bio), the band at 123–125bp was cut out from the gel, and DNA purified after its elution from the gel pieces. The PCR product from each sample was analyzed on an Agilent Bioanalyzer 2100. Equal concentrations of PCR products from all the samples were mixed and applied to Illumina Genome Analyzer II sequencing.

### Sequencing

Purified and mixed PCR products were applied to cluster formation on the flowcell of the Illumina Genome Analyzer II. Sequencing reactions used GEX (DpnII) primer following the instructions of the manufacturer.

### Data analysis

Sorting of sequence reads based on index sequences and the subsequent extraction of sequence tags from reads was conducted using a script written in Perl. Tag profiling data (list of tag sequences and their count) was registered in NCBI Gene Expression Omnibus (its accession number is GSE20682).

Unigene databases were downloaded from the FTP site in NCBI (ftp.ncbi.nih.gov/repository/UniGene). *Magnaporthe grisea* whole genome draft sequences were downloaded from Broad Institute (www.broadinstitute.org/annotation/genome/magnaporthe_grisea/Downloads.html). Arabidopsis thaliana RefSeq database was downloaded from the FTP site of The Arabidopsis Information Resources (ftp.arabidopsis.org).

Arabidopsis tag mapping was performed with Novoalign software (Novocraft Technologies).

## Supporting Information

Figure S1 Distribution of extracted tag length. Tags were extracted from sequence reads of sample, and number of total and unique tags from 26 to 31 bases were estimated.
(0.12 MB PPT)

Figure S2 Comparison of tag abundance between HT-SuperSAGE and original SuperSAGE after removal of tags harboring homopolymer sequences and tags, which did not completely matched rice and Magnaporthe grisea genome sequences from dataset in Figure 3. Criteria of tag removal was described in the text. (panel A for sample f and panel B for sample l).
(0.10 MB PPT)

Figure S3 Comparison of tag count among three replicated rice shoot samples (sample d, e, and f). Red arrow indicates the tag “CATGACAAGTTTTTGTTAATAATAAT”.
(0.57 MB PPT)

Table S1 Summary of BLAST searching of the top 10 most abundant tags in each sample.
(1.56 MB TIF)

Table S2 Total number of analyzed tags by HT-SuperSAGE and original SuperSAGE.
(1.56 MB TIF)

Table S3 Counts of tags containing homopolymer sequences.
(1.56 MB TIF)

Table S4 Frequency of tags, which may have been caused by errors.
(1.56 MB TIF)

Table S5 Transcript amount of Arabidopsis genes analyzed by tag extraction using three different anchoring enzymes (NlaIII, DpnII, BfaI).
(3.45 MB XLS)
