# Supplementary material for: High-Throughput SuperSAGE for Digital Gene Expression Analysis of Multiple Samples Using Next Generation Sequencing
Source: PLoS One. 2010 Aug 6;5(8):e12010. doi: 10.1371/journal.pone.0012010 (PMC2917361; doi:10.1371/journal.pone.0012010)
Supplement: Figure S1 — Distribution of extracted tag length. Tags were extracted from sequence reads of sample, and number of total and unique tags from 26 to 31 bases were estimated. (0.12 MB PPT) [file pone.0012010.s001.ppt]

## Slide 1
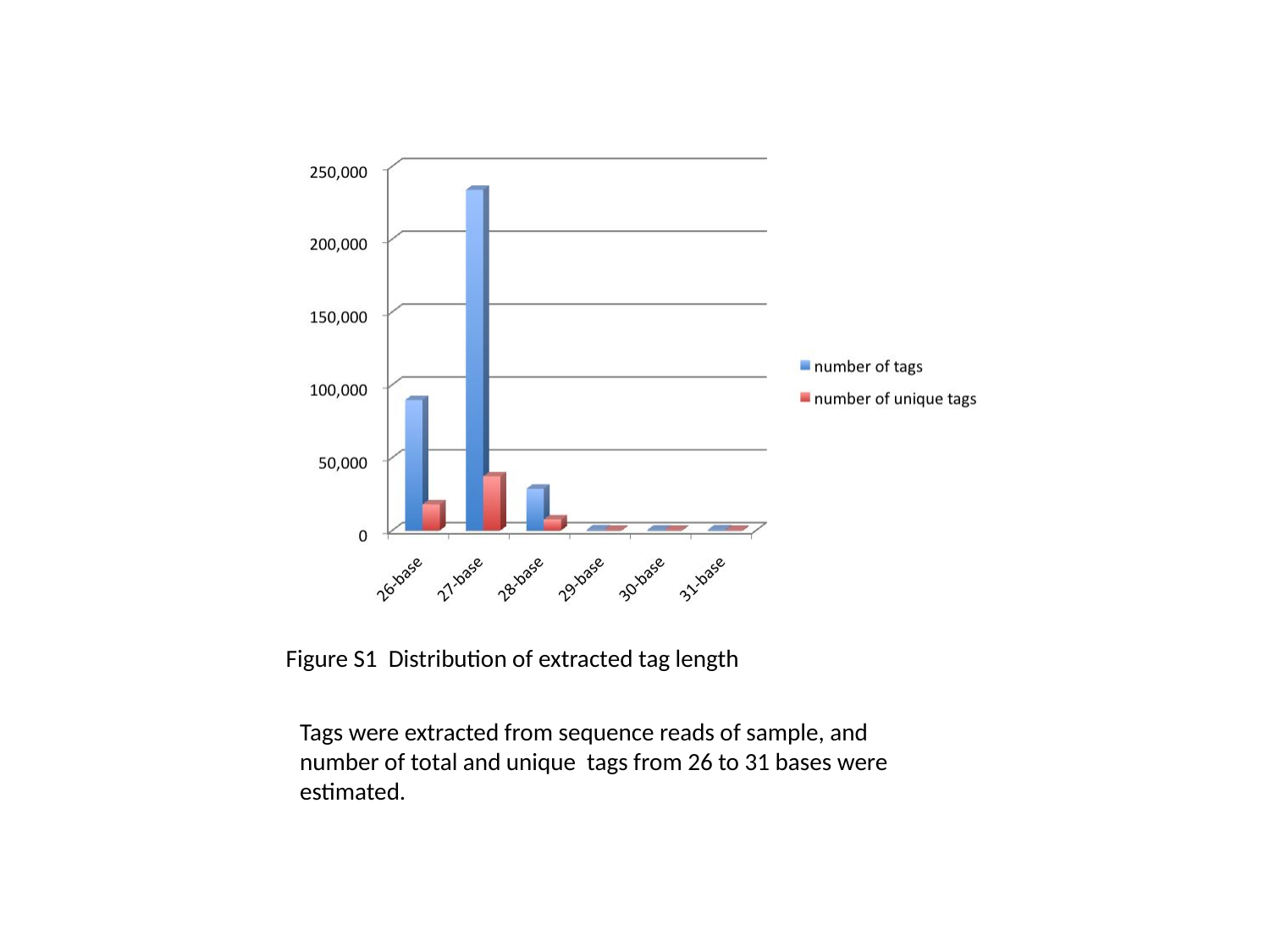

Figure S1 Distribution of extracted tag length
Tags were extracted from sequence reads of sample, and number of total and unique tags from 26 to 31 bases were estimated.
