# Supplementary material for: High-Throughput SuperSAGE for Digital Gene Expression Analysis of Multiple Samples Using Next Generation Sequencing
Source: PLoS One. 2010 Aug 6;5(8):e12010. doi: 10.1371/journal.pone.0012010 (PMC2917361; doi:10.1371/journal.pone.0012010)
Supplement: Figure S2 — Comparison of tag abundance between HT-SuperSAGE and original SuperSAGE after removal of tags harboring homopolymer sequences and tags, which did not completely matched rice and Magnaporthe grisea genome sequences from dataset in Figure 3. Criteria of tag removal was described in the text. (panel A for sample f and panel B for sample l). (0.10 MB PPT) [file pone.0012010.s002.ppt]

## Slide 1
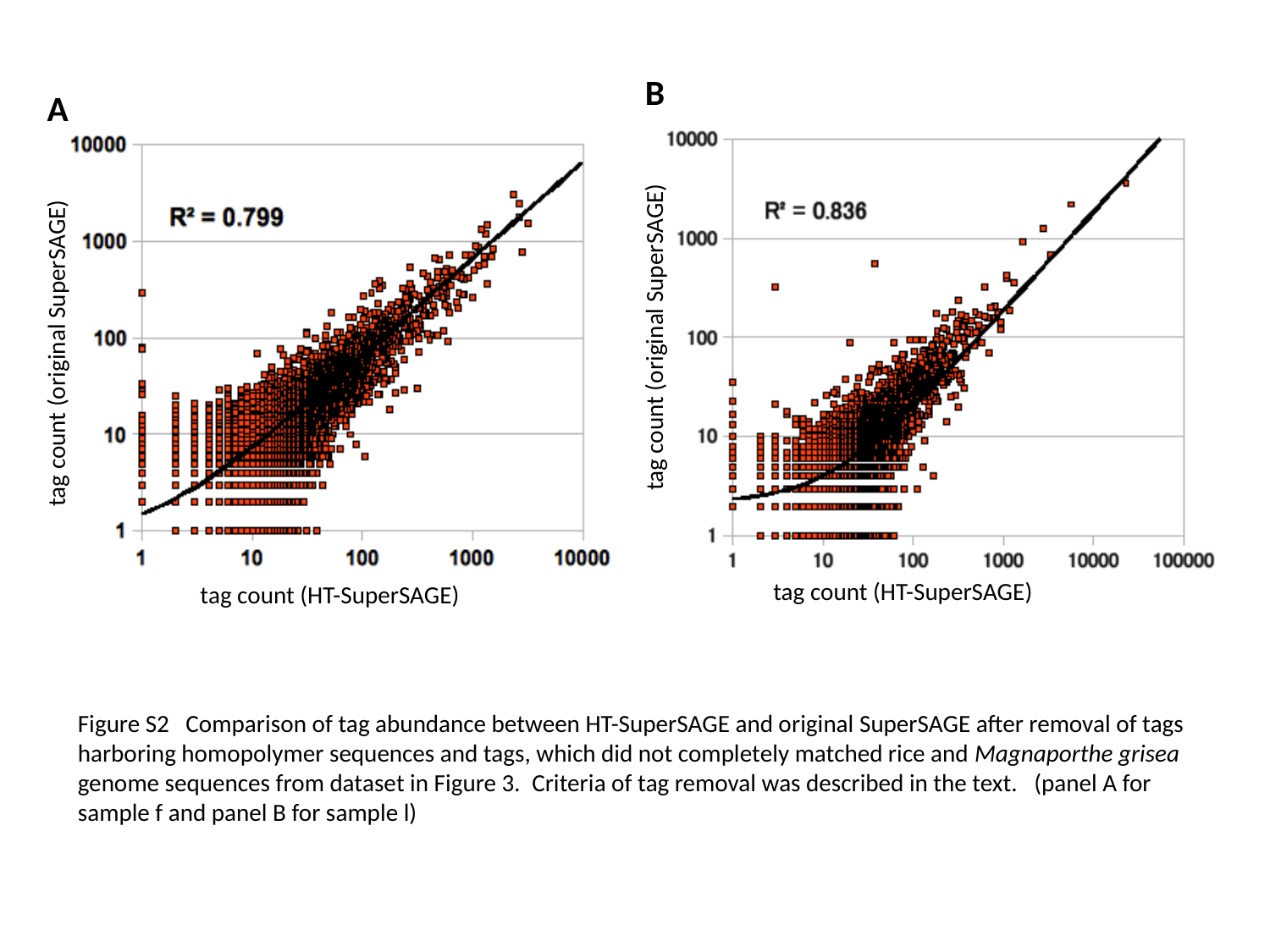

B
A
tag count (original SuperSAGE)
tag count (original SuperSAGE)
tag count (HT-SuperSAGE)
tag count (HT-SuperSAGE)
Figure S2 Comparison of tag abundance between HT-SuperSAGE and original SuperSAGE after removal of tags harboring homopolymer sequences and tags, which did not completely matched rice and Magnaporthe grisea genome sequences from dataset in Figure 3. Criteria of tag removal was described in the text. (panel A for sample f and panel B for sample l)
