# Supplementary material for: High-Throughput SuperSAGE for Digital Gene Expression Analysis of Multiple Samples Using Next Generation Sequencing
Source: PLoS One. 2010 Aug 6;5(8):e12010. doi: 10.1371/journal.pone.0012010 (PMC2917361; doi:10.1371/journal.pone.0012010)
Supplement: Figure S3 — Comparison of tag count among three replicated rice shoot samples (sample d, e, and f). Red arrow indicates the tag “CATGACAAGTTTTTGTTAATAATAAT”. (0.57 MB PPT) [file pone.0012010.s003.ppt]

## Slide 1
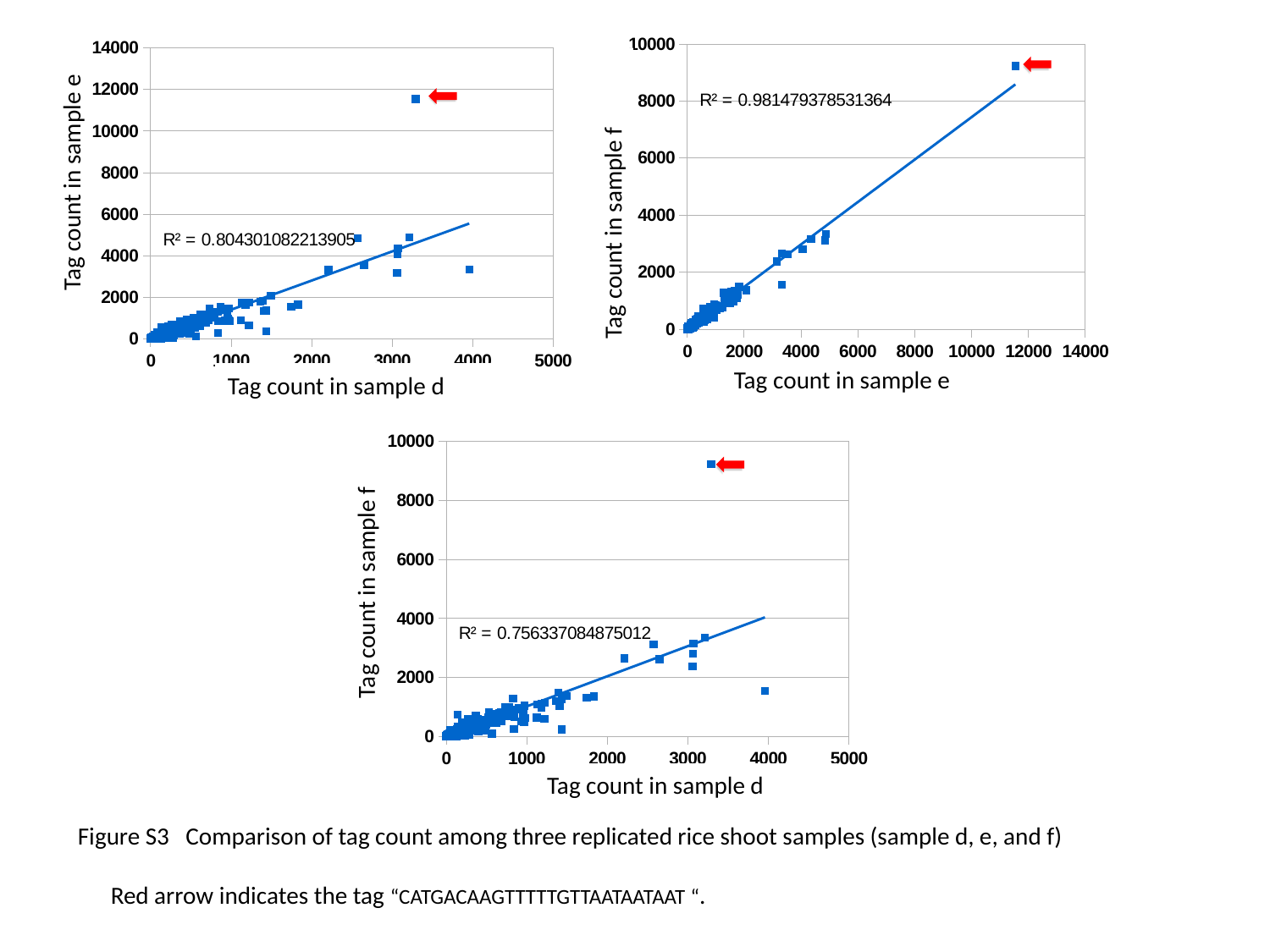

### Chart
| Category | 列 D |
|---|---|
### Chart
| Category | 列 C |
|---|---|
Tag count in sample e
Tag count in sample f
Tag count in sample e
Tag count in sample d
### Chart
| Category | 列 D |
|---|---|
Tag count in sample f
Tag count in sample d
Figure S3 Comparison of tag count among three replicated rice shoot samples (sample d, e, and f)
 Red arrow indicates the tag “CATGACAAGTTTTTGTTAATAATAAT “.
