# Supplementary figures and images for: High-Throughput SuperSAGE for Digital Gene Expression Analysis of Multiple Samples Using Next Generation Sequencing
Source: PLoS One. 2010 Aug 6;5(8):e12010. doi: 10.1371/journal.pone.0012010 (PMC2917361; doi:10.1371/journal.pone.0012010)

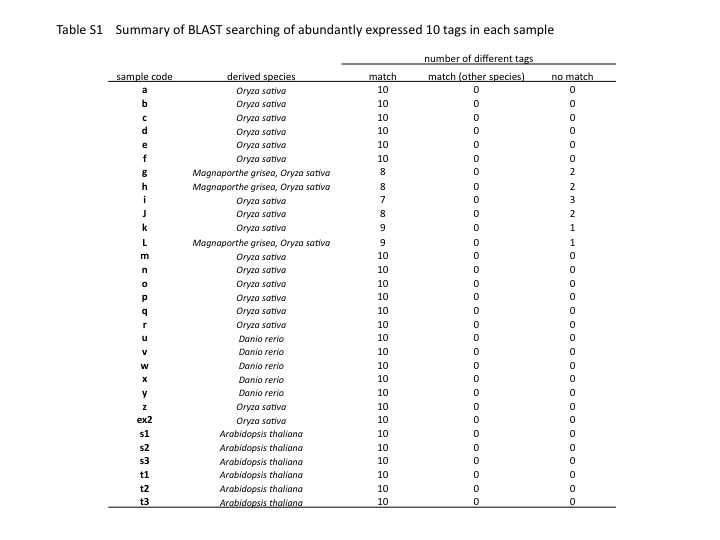

Supplement: Table S1 — Summary of BLAST searching of the top 10 most abundant tags in each sample. (1.56 MB TIF) [file pone.0012010.s004.tif]

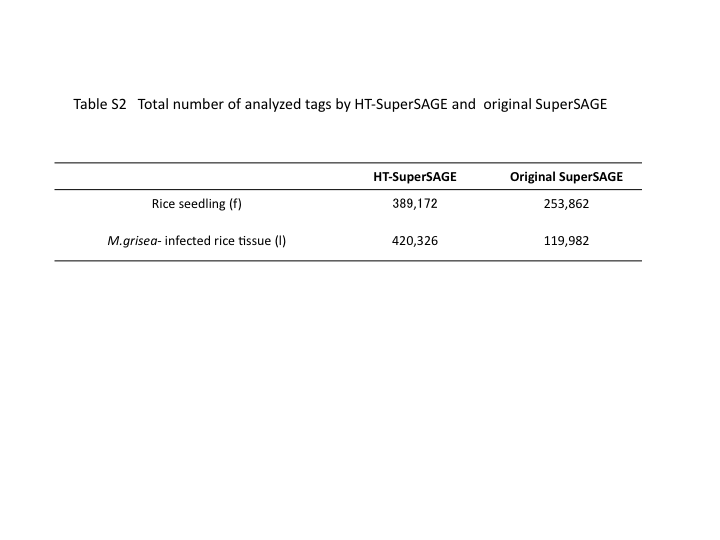

Supplement: Table S2 — Total number of analyzed tags by HT-SuperSAGE and original SuperSAGE. (1.56 MB TIF) [file pone.0012010.s005.tif]

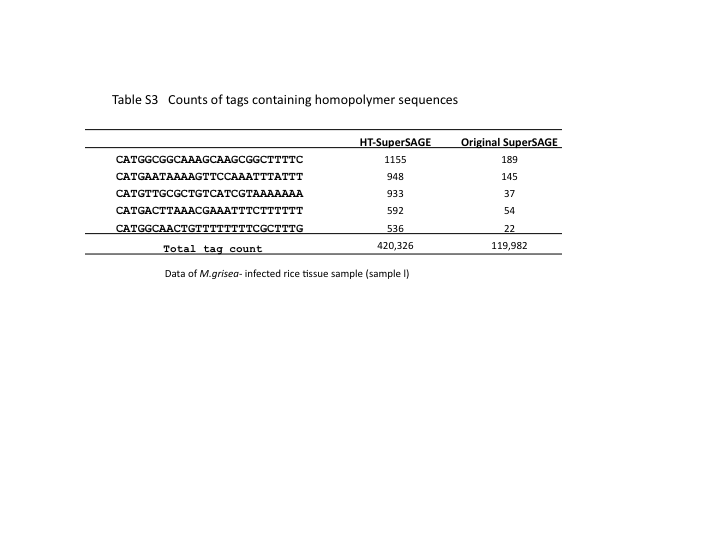

Supplement: Table S3 — Counts of tags containing homopolymer sequences. (1.56 MB TIF) [file pone.0012010.s006.tif]

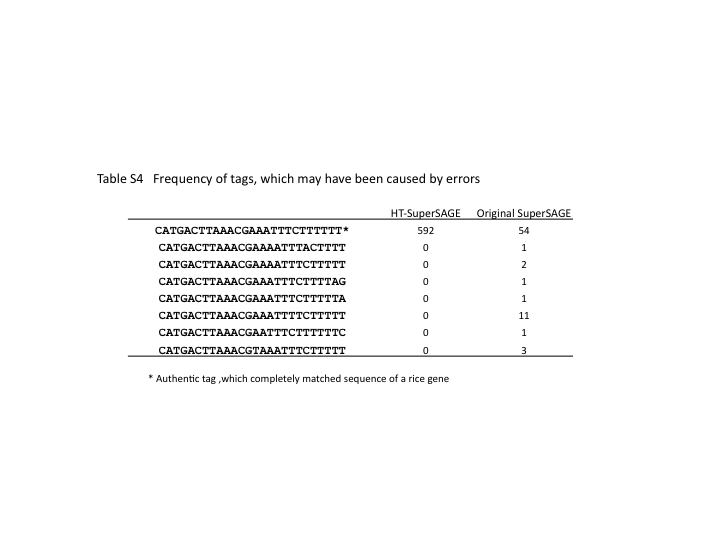

Supplement: Table S4 — Frequency of tags, which may have been caused by errors. (1.56 MB TIF) [file pone.0012010.s007.tif]
